# Dysregulation of NF-kB in glandular epithelial cells results in Sjögren’s-like features

**DOI:** 10.1371/journal.pone.0200212

**Published:** 2018-08-01

**Authors:** Xiaoyan Wang, Abeer Shaalan, Silvia Liefers, Julie Coudenys, Dirk Elewaut, Gordon B. Proctor, Hendrika Bootsma, Frans G. M. Kroese, Sarah Pringle

**Affiliations:** 1 Department of Rheumatology and Clinical Immunology, University Medical Centre Groningen, University of Groningen, Groningen, The Netherlands; 2 Division of Mucosal and Salivary Biology, King’s College London Dental Institute, King’s College London, London, United Kingdom; 3 Department of Rheumatology, Ghent University Hospital, Ghent, Belgium; 4 Unit Molecular Immunology and Inflammation, VIB Center for Inflammation Research, Ghent University, Ghent, Belgium; University of Bergen, NORWAY

## Abstract

The autoimmune disease primary Sjögren’s syndrome (pSS) is characterized by hypofunction of the salivary glands (SGs), the cause of which is not correlated to lymphocytic SG infiltration, as prevailing dogma often states. We knocked out the NF-κB proinflammatory pathway inhibitor A20 in keratin14^+^ epithelial cells, to investigate if immune activated epithelial cells are capable of initiating pSS SG hallmarks. We show that immune activated epithelial cells can cause T cell dominated leukocytic infiltration and immune foci development of the SGs, reflecting the early clinical picture. Infiltrating leukocytes invaded striated ducts, similar to early stage lymphoepithelial lesions observed clinically. Expression of proinflammatory cyto-/chemokines IFNɣ, TNFα, IL-6, CXCL10 and CXCL13 increased in A20^-/-^ SGs, and functionally both volume and mucin 10 content of whole stimulated saliva from A20^-/-^ mice was significantly reduced. Epithelial cells may therefore represent the initial trigger for pSS SG pathologies, as opposed to simple reactionaries to pre-existing stimuli.

## Introduction

The origins of hyposalivation in the autoimmune disease primary Sjögren’s syndrome (pSS) have remained an enigma for many years. Presence of lymphocytic foci in salivary glands (SGs) of pSS patients has been well documented, and were assumed to represent the causative agents underpinning hyposalivation in pSS. Recently however, several studies have shown that hyposalivation begins substantially earlier than the development of lymphocytic infiltration and immune foci in patient SGs, and that the two cannot be correlated [1].

In the current study, we test the hypothesis that the SG epithelium, namely the ductal cells, play a salient role in SG pathology development in pSS. Ductal epithelial cells of the SG have been implicated in SG pathogenesis in pSS in numerous studies [2]. Expression of MHC Class II, CD80/86 and CD54 highlight their potential as antigen presenting cells. Epithelial cells of pSS patients express the toll-like receptors (TLRs) 1,2,3,4 and 7, produce IL-6 when subjected to TLR stimulation, are markedly more sensitive to TLR3-induced apoptosis, and can be induced to undergo anoikis following TLR3 ligation by double-stranded RNA [3,4]. SG epithelial cell apoptosis has also be suggested to follow both SG epithelial cell exposure to pSS-associated autoantibodies and enhanced level of TNFα activity [5]. TNFα expression is not only increased in the SG as a whole in pSS, but also specifically in epithelial cells [6]. Several studies have examined SG epithelial cell response to poly(I:C), IFNα, IFNɣ and TNFα stimulation, with authors reporting high levels of BAFF, CXCL9 and CXCL10 production [7,8].

The above studies paint the epithelium as a central character in pSS SG pathology, and collectively show that it produces important pro-inflammatory molecules and receives immune signals. Despite all these findings it remains to be shown whether pSS can be initiated by chronic immune activation of the glandular epithelium. Here we use a mouse model where A20 is knocked out under control of the keratin 14 (KRT14) promoter. In the mouse SG, KRT14 expression is limited to the basal cells of the striated ducts and excretory ducts, intercalated ductal cells, and myoepithelial cells. The A20 protein is an inhibitor of NF-κB signaling, one of the classical pro-inflammatory signaling pathways. Knocking out A20 thus promotes a pro-inflammatory environment in KRT14^+^ cells. We present a model focusing on the immune capabilities of epithelial cells themselves as triggers of immune reactions rather than targets, and show that immune activation of epithelial cells of the SG is enough to generate major hallmarks of pSS SG pathology.

## Results

### Intrinsically activated epithelial cells cause T-cell rich lymphocytic infiltration of salivary glands

**Fig 1:**
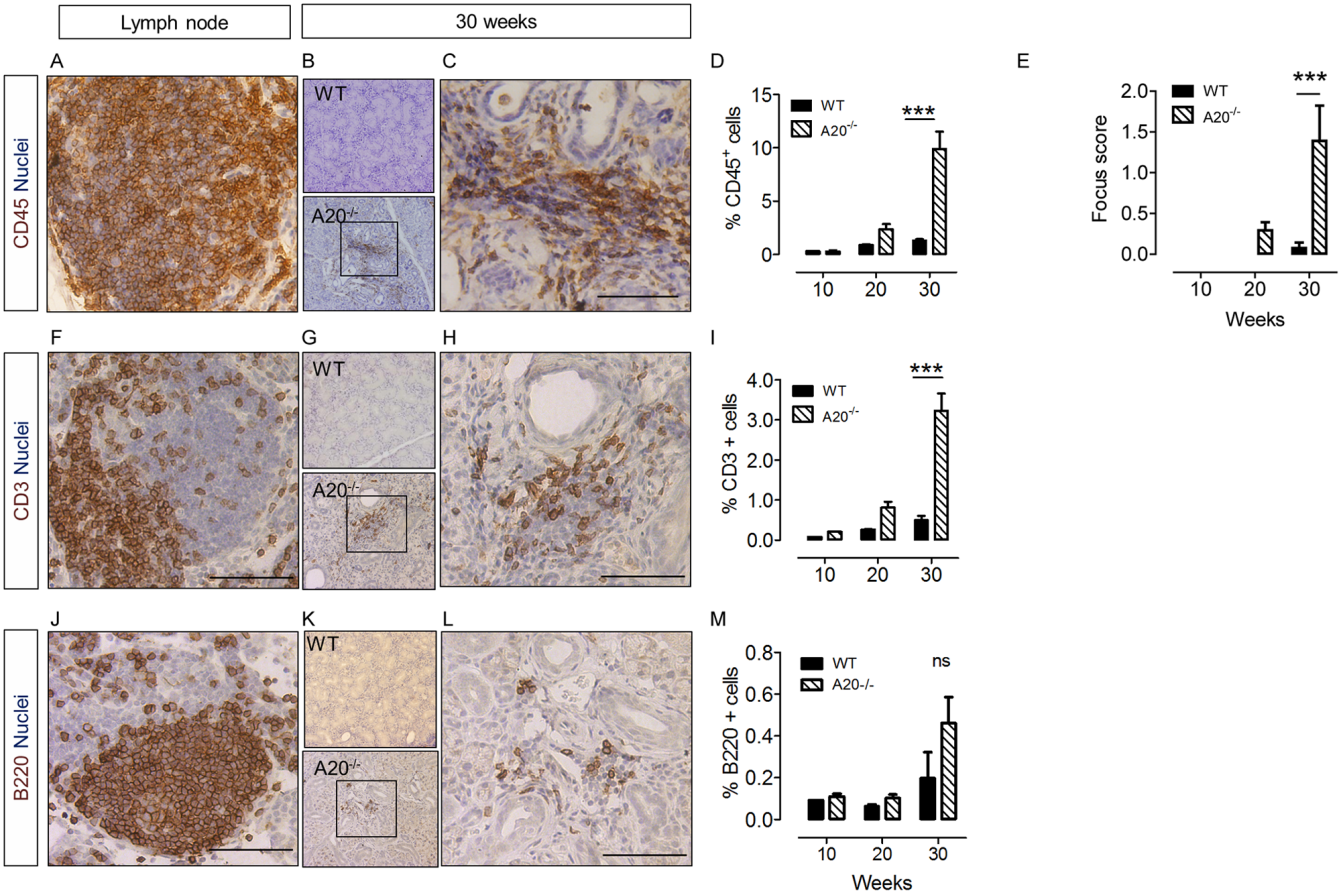
Activated epithelial cells cause lymphocytic salivary gland infiltration. A) Lymph node positive staining control for CD45 immunostaining. B) Low resolution immunostaining for CD45, denoting all leukocytes, in WT and A20^-/-^ mice at 30 weeks of age. C) High resolution of boxed area in B showing foci of CD45^+^ cells. D) Quantification of proportion of CD45^+^ cells in WT and A20^-/-^ mice submandibular salivary glands mice at 10, 20 and 30 weeks of age. E) Quantification of foci, defined as > 50 CD45^+^ cells together, per 4mm^2^ of submandibular SG tissue. *n* = 6 biological replicates for all groups, apart from 10 week time point, where *n* = 1 and 3 for WT and A20^-/-^ respectively. *** = p < 0.001, Two Way ANOVA. F) Lymph node positive staining control for CD3 T cell immunostaining. G) Low resolution microscopy of CD3 immunostaining, denoting T cells, in WT and A20^-/-^ mice at 30 weeks. H) High resolution of boxed area in F. I) Quantification of proportion of T cells in WT and A20^-/-^ mice at submandibular salivary glands at 10, 20 and 30 weeks of age. J) Lymph node positive staining control for B220 immunostaining. K) Low resolution immunostaining images of B220, denoting B cells, in WT and A20^-/-^ mice at 30 weeks of age. L) High resolution of boxed area in J. M) Quantification of proportion of B cells in WT and A20^-/-^ mice submandibular glands at 10, 20 and 30 weeks of age. For all quantification data *n* numbers are as panel E. *** = p < 0.001.

In the present mouse model A20 was knocked out in KRT14-expressing cells of the murine submandibular salivary glands, found in the excretory ducts, basal striated duct cells, intercalated duct cells and myoepithelial cells (Fig B in S1 File). Immunohistochemical staining demonstrated presence of CD45^+^ leukocytic infiltrate, in A20^-/-^ mice. CD45^+^ cells were located as foci (more than 50 CD45^+^ cells together) positioned close to striated ducts, and also as dispersed cells (Fig 1B and 1C). The percentage of CD45^+^ cells increased in A20^-/-^ mice from 10 to 30 weeks to a maximum of 9.91% (± 1.62 SEM) of total cells present in sections, and was significantly greater than that in WT controls (Fig 1D). A20^-/-^ mice contained an average of 1.39 ± 0.43 SEM foci / 4 mm^2^ tissue at 30 weeks of age, significantly greater than those in WT mice (0.07 ± 0.08 SEM foci / 4 mm^2^) (Fig 1E). CD3^+^ T cells were also found in foci and dispersed through the gland, and comprised a significantly greater proportion of total SG cells at 30 weeks in A20^-/-^ mice, compared to WT controls (3.23% ± 0.43 SEM compared to 0.50% ± 0.11 SEM; Fig 1F–1I). B220^+^ cells B cells were detected only in immune foci, and not dispersed through the gland (Fig 1J–1L). Although displaying a trend for increase in B cell proportion over time, the difference was not significant compared to controls (Fig 1M). There was no difference detected in the degree of leukocytic, T cell or B cell infiltration between male and female A20^-/-^ mice (Figure C in S1 File). All histological images shown in Fig 1 are from male mice.

### Intrinsically activated epithelial cells cause invasion of striated ducts

**Fig 2:**
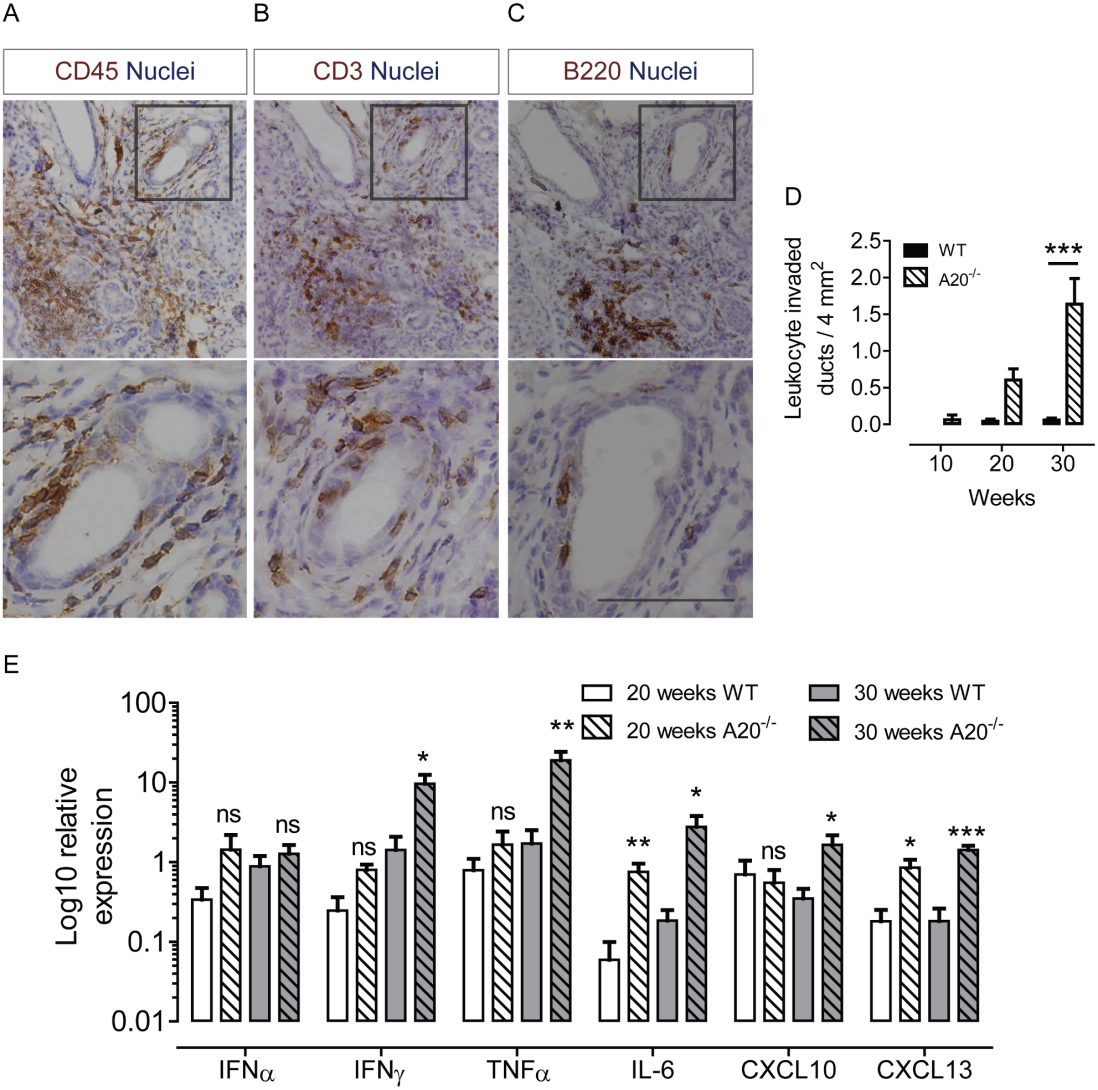
Activated epithelial cells cause leukocyte striated duct invasion and local increased cytokine production. A) Leukocytic (CD45^+^) invasion of a striated duct in A20^-/-^ mice at 30 weeks of age. B) CD3^+^ T cell invasion of a striated duct in serial section to A. C) B220^+^ B cell invasion of a striated duct in serial section to A. Second panels in A-C are high resolution images of inset boxes. D) Quantification of invaded striated ducts frequency per 4 mm^2^ of glandular tissue. *** = p< 0.001, Two Way ANOVA. E) Relative expression of the pro-inflammatory cytokines (IFNα, IFNɣ, TNFα and IL-6) and pSS-associated chemokines (CXCL10, CXCL13) in A20^-/-^ mice at 20 and 30 weeks of age. *n* ≥ 5 per group. * p < 0.05, ** p < 0.01, *** p < 0.001, student’s *t*-test.

Lymphoepithelial lesions, whereby lymphocytes are found between ductal epithelial cells, are characteristic of pSS SG pathology and lead to a higher risk of potentially fatal non-Hodgkin’s lymphoma. In our mouse model, leukocyte invasion of striated ducts was observed in A20^-/-^ mice at 30 weeks (Fig 2A). Invaded striated ducts were quantified per 4 mm^2^ of tissue (Fig 2D). Striated duct invasion was rarely observed in control mice. In A20^-/-^ mice at 30 weeks of age, invaded ducts were observed at a statistically significant higher frequency than controls. Immunostaining for CD3 and B220 revealed that T cells, and occasional B cells were observed invading striated ducts (Fig 2B and 2C).

### Intrinsically activated epithelial cells cause increase in local pro-inflammatory chemokine and cytokine production

At 20 weeks of age, and thus before significant appearance of immune foci, qPCR analysis demonstrated an increase in expression of the pro-inflammatory cytokines IFNα, IFNɣ, TNFα IL6 and the B lymphocyte attractant CXCL13 in the SGs of A20^-/-^ mice compared to controls. IFNɣ, TNFα and IL-6, CXCL13 and the leukocyte attractant CXCL10 were further significantly upregulated in SGs of 30 week old A20^-/-^ mice compared to WT controls (Fig 2E). These data suggest that immune activation of dysregulated epithelial cells culminating in augmented NFκB pathway activity is sufficient to predispose the salivary gland for the development of an inflammatory immune milieu. In order to examine the possibility that intrinsically activation of epithelial is sufficient to induce autoantibody formation, we examined serum from peripheral blood of A20^-/-^ mice, for the presence of pSS-associated autoantibodies, in comparison to levels observed in control mice. Autoantibodies were not detected in A20^-/-^ serum at a higher titer (score) or proportion of mice than WT control mice (Fig D in S1 File), suggesting that epithelial cell activation alone is not sufficient for systemic disease initiation.

### Intrinsically activated epithelial cells cause a reduction in saliva production and salivary mucin content

**Fig 3:**
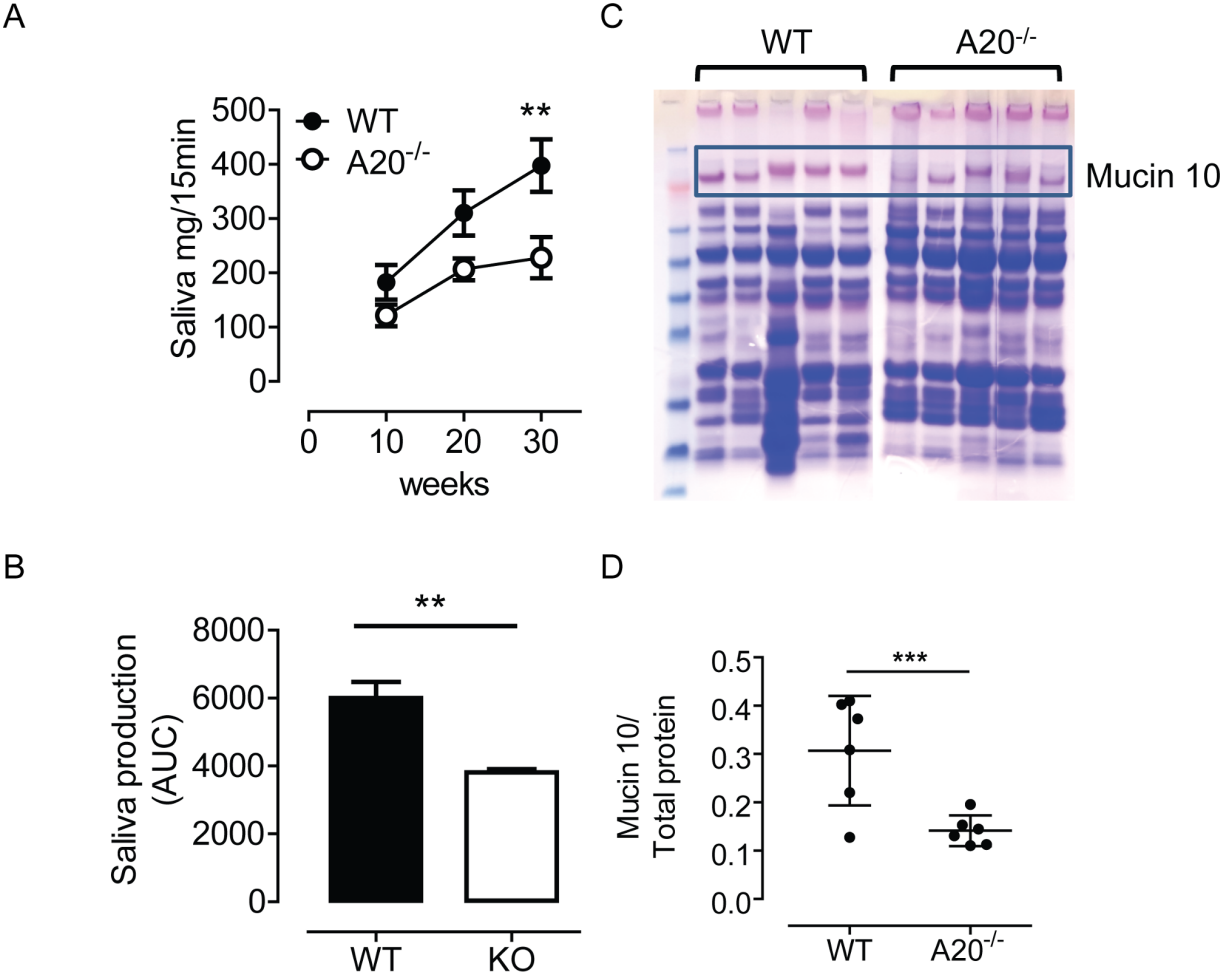
Activated epithelial cells lead to reduction in both volume of saliva produced and Mucin 10 present in saliva. A) Whole stimulated saliva produced by A20^-/-^ mice and WT littermate controls. *n* = 6 mice per group per time point Two Way ANOVA testing showed significant difference at the 30 week time point. ** p < 0.01 B) Area under curve analysis of saliva production in A. Student’s *t*-test was performed for statistical analysis, ** *p* < 0.01. C) Coomassie and Periodic Acid Shift stained gel of whole stimulated saliva from WT and A20^-/-^ mice. Position of bands representing Mucins 10 is shown. Each lane represents saliva from a separate biological replicate. D) Quantification amount of Mucin 10 in saliva, relative to total protein. Each data point represents a separate biological replicate. *** *p* < 0.001, student’s *t*-test.

Whole stimulated saliva production was lower in A20^-/-^ mice compared to wildtype littermate controls, when measured at 10, 20, and 30 weeks and adjusted for pilocarpine dose (Fig 3A, Fig E in S1 File and methods for pilocarpine correction). Area under the curve analysis confirmed the significant nature of this difference (Fig 3B). As detected by a combination of Coomassie/PAS stained gels and mass spectrometry, saliva from A20^-/-^ mice contained significantly less mucin 10 (MUC10), associated with murine submandibular gland acinar cells, than WT controls (Fig 3C and 3D).

## Discussion

Evaluation of salivary glands for criteria suggestive of pSS generally takes into account presence of immune foci, lymphoepithelial lesions, germinal centers and an influx of IgG-producing plasma cells. In contrast to existing studies, we employ a cell intrinsic signal specific to the epithelium, to explore whether with such an approach initiation of Sjögren’s syndrome-like pathologies is possible. This is in stark opposition to the commonly employed tactics of extrinsic administration of pathway stimulants or genetic manipulation of cells belonging to the immune system repertoire, used in other models [9].

The present study demonstrates that activation of the NF-κB pathway in KRT14^+^ epithelial cells results in features that resemble the early histopathological phases of pSS. These features include presence of both T-cell dominated periductal infiltrates organized in foci, and of non-occluded lymphoepithelial lesions. Elevated mRNA levels of pro-inflammatory cytokines IFNα, IL-6, TNFα, IFNɣ and chemokines CXCL10 and CXCL13 in the glandular tissue demonstrated in A20^-/-^ mice also strongly suggest mimicry of the immune milieu found in early human pSS. IFNα in particular, a hallmark of pSS, was upregulated at 20 weeks of A20^-/-^ mice, notably earlier than significant presence of immune foci, consistent with a role in the initiation phase of pSS. These data suggest that dysregulated activation of epithelial cells, resulting in prolonged production of proinflammatory cytokines is sufficient to prime the salivary glands for development of an inflammatory, pSS-like immune milieu. Interestingly, saliva production also begins to deteriorate at 20 weeks of knockout mouse age, in line with clinical data suggesting that hyposalivation in pSS is not correlated with presence of immune foci [1]. Expression of the pSS-associated homeostatic B-cell attractant chemokine CXCL13 was significantly upregulated already at the 20 week time point. The sum presence of all of these key, pSS-associated cytokines strongly suggests the establishment of a phenotype mirroring the early clinical picture, using A20^-/-^ solely in epithelial cells.

Full recapitulation of later pSS phases may require further activation and/or damage of epithelial cells, possibly in combination with hyperactivated, autoimmune prone, B cells leading ultimately to autoantibody production. Likely triggers, such as for example micro-organism induced stimulation of epithelial toll-like receptors and cytosolic sensors, result initially in activation of the NF-κB pathway, which then drives the inflammatory process seen in the salivary glands of the A20^-/-^ mice. In the absence of its natural inhibitor A20, chronic dysregulation of NF-κB function will ensue, and result ultimately in ongoing inflammation, similar to what is observed in pSS. Histopathologically, these more advanced stages will be characterized by predominance of B-cells within the foci, presence of germinal centers, occluded lymphoepithelial lesions and influx of IgG producing plasma cells.

Our strategy for investigation of epithelial cell ability to initiate pSS-like traits is based around constitutive activation of the canonical NF-κB pathway in KRT14^+^ cells. A20 blocks the ubiquitination of the serine-threonine kinases RIP and IκB, components of the canonical NF-κB signaling pathway. As evidenced by elevated mRNA levels of the canonical NF-κB downstream targets IFNα, TNFα and IL-6 in the whole salivary gland, the NF-κB pathway is indeed in an active state. Ductal epithelial cells from patients with pSS have been demonstrated to express IL-6 and TNFα, suggesting that direct results of NF-κB activation in KRT14^+^ cells could be their proinflammatory cytokine expression [6]. Together with several other factors, NF-κB signaling is also involved in expression of IFNɣ. IFNɣ is a major cytokine present in pSS salivary glands [6], and in concert with other cytokines, for example TNFα, can upregulate the expression of the chemokine CXCL10 by epithelial cells [8]. In our A20^-/-^ study, not only were TNFα, IFNɣ and IL-6 highly upregulated, but also CXCL10 as well as the B cell attractant CXCL13, at the 30 week time point. At 20 weeks, CXCL10 expression was not upregulated, suggesting perhaps that CXCL10 expression requires augmented IFNɣ signalling. In the early phases of pSS pathology particularly, CXCL10 plays a major role in the attraction of CXCR3-expressing cells, mainly CD4^+^ Th1 T cells, but potentially also T-bet^+^ B cells [10,11]. These recruited cells may further contribute to the IFNɣ signature of the gland. In reinforcement of our data, NZB/W F1 mice injected with the TLR3 agonist poly(I:C) stimulating NF-κB activation globally in the mouse, demonstrated sialodentitis and hyposalivation, and also CXCL10 expression [12]. Furthermore, these authors found that in the early stages after poly(I:C) administration, most lymphoid cells in the immune foci were CD11c^+^ dendritic cells and CD4^+^ T cells, followed by NK-cells, whereas B-cells were not significantly increased compared to controls [12]. In our study, most cells in the immune infiltrates were also non-T, non-B cells. According to Nandula *et al*, this cohort of cells potentially represents NK and dendritic cells, cells endowed with ability to augment IFNɣ production in the salivary glands and further the inflammatory environment. Expression of the chemokine CXCL13 is largely under the control of the non-canonical NF-κB pathway. This pathway is not directly regulated by A20. The observed early expression of CXCL13 is thus an indirect consequence of the A20^-/-^ and may be attributable perhaps to CD11c^+^ dendritic cells. Immune cell culprits aside, CXCL13 expression may also emanate from other stromal cell types, their exact identity remaining to be defined. The increased expression of CXCL13 is promising in terms of the ability of activated epithelial cells to indirectly set the stage for the B cell hyperactivity-associated disease pSS. CXCL13 levels have been shown to be enhanced in pSS patients [13].

Infiltrating leukocytes potentially attracted by the concerted action of TNFα, IL6 and IFNɣ, CXCL10 and CXCL13 were found to be organized into immune foci, localized to the striated ducts. A second feature of the pSS salivary gland in relation to infiltration is the presence of lymphoepithelial lesions, whereby B cells invade striated ducts, culminating in hyperplasia of epithelial cells. In humans, signals driving epithelial cell hyperplasia appear to emanate from these invading B cells. In our model, leukocytes were observed infiltrating the striated ducts, but in absence of accompanying epithelial cell hyperplasia, a logical explanation for which would be the B-cell poor nature of our early pSS-like infiltration.

On a last clinical note, pSS presents with a consistent 9:1 ratio of female to male patients. Chronic immune activation of epithelial cells by knockout of A20 did not result in a sex-based dimorphism in terms of pSS-like symptoms, very much in line with the NOD, *Aec1/Aec2* and *IQI/Jic* systems for pSS mimicry (reviewed in [9]). The female preponderance of pSS may be linked with altered local intracrine metabolism of sex steroids, a further factor to consider incorporating into future versions of our mouse model.

Finally, homeostasis of the SG is maintained by proliferation and differentiation of tissue resident stem cells (salivary gland stem cells; SGSCs) into saliva producing acinar cells [14]. SGSCs are resident in the ducts, KRT14^+^ in nature, and are thus subject to A20 knockout, in this mouse model. A20 knockout is permanent once induced, and so consequently acinar cell progeny of KRT14^+^ SGSCs will also lack the A20 NF-κB inhibitor. Both saliva volume and MUC10 content of A20^-/-^ mice saliva was reduced in the 30 week mice, indicating defects in acinar cell functional capabilities. Whether these functional attenuations arise from inherited (SGSC-derived) acinar cell A20 knockout status, or the independent influence of NF-κB on acinar cell secretory power, or concerted action of the two, requires further investigation.

In the current study, we sought to investigate the hypothesis that immune activation of epithelial cells is enough to generate the majority of the pathological features associated with the salivary gland in pSS. We have shown for the first time to our knowledge that epithelial cells lie at the heart of initiation of salivary pathology in a pSS mouse model.

## Materials and methods

### Mice breeding and genotyping

A20^-/-^ parental mice were kindly provided by Geert van Loo, at the Flemish Institute for Biotechnology (VIB). To generate A20^-/-^ mice under the control of the K14 promoter (K14Cre^wt/ko^_A20^wt/fl^), female K14Cre^wt/wt^_A20^wt/fl^ mice were cross-bred with male K14Cre^wt/ko^_A20^wt/fl^ mice. To generate these parental strains, A20^fl/fl^ mice as published by Kool *et al* (2011) were crossed with K14Cre mice. A total of 40 mice were used for this study. Fourteen mice (8 WT and 6 A20^-/-^) were maintained until the 30 week time point. Of remaining mice, 9 (2 WT, 7 A20^-/-^) were sacrificed for histological analysis at 10 weeks, and the final 17 mice (8 WT, 9 A20^-/-^) at 20 weeks. Both male and female mice were used. Mice were housed under conventional conditions and fed *ad libitum* with food pellets (RMH-B, Hope Farms, B.V.) and water. All experiments were approved by the Ethical Committee on animal testing of the University of Groningen. All mice were sacrificed under isoflurane anesthesia using cervical dislocation.

### Saliva measurements, immunohistochemistry, saliva protein analysis and qPCR

Standard protocols were used for all these techniques. Extended methods for each can be found in S1 File.

## Supporting information

S1 File Table A. Primers for genotyping of K14Cre^wt/fl^_A20^wt/fl^ mice and for qPCR analysis of cytokine/chemokine production. Abbreviations: WT = wildtype; KO = knockout; REC = recombinase. Figure A. A20^-/-^ mice genotyping example. Representative genotyping by PCR for identification of *K14Cre*^*wt/fl*^*_A20*^*wt/fl*^ mice. Expected sizes of products are shown. Figure B Confirmation of localization of KRT14+ cells. Immunohistochemical stainings for expression of KRT14, showing localization in basal layers of excretory, striated, in intercalated ducts and in myoepithelial cells. Figure C No difference was observed in salivary gland infiltration between male and female A20^-/-^ mice. Left panel: Quantification of proportion of total cells staining positive for CD45 in male and female WT and A20^-/-^ mice. *n* = 3 mice per group per time point. Middle panel: Quantification of proportion of CD3^+^ cells as proportion of total cells in male and female WT and A20^-/-^ mice. Right panel: Quantification of proportion of B220^+^ cells as proportion of total cells in male and female WT and A20^-/-^ mice. Figure D No autoantibodies were detected in A20^-/-^ serum. Summary of ANA autoantibody score from WT and A20^-/-^ mice at 30 weeks of age. For scoring system, please see Methods. Figure E Significant weight loss of A20^-/-^ mice necessitates a correction for pilocarpine dose. Top panel: Weights of *K14Cre*^*wt/fl*^*_A20*^*wt/fl*^ mice compared to WT littermate controls. Bars = S.D. *n* = ≥ 6 mice per group per time point. Bottom panel: Example corrections of pilocarpine measurements.
(DOCX)
