# Supplementary material for: Dysregulation of NF-kB in glandular epithelial cells results in Sjögren’s-like features
Source: PLoS One. 2018 Aug 1;13(8):e0200212. doi: 10.1371/journal.pone.0200212 (PMC6070175; doi:10.1371/journal.pone.0200212)
Supplement: S1 File — Table A. Primers for genotyping of K14Crewt/fl_A20wt/fl mice and for qPCR analysis of cytokine/chemokine production. Abbreviations: WT = wildtype; KO = knockout; REC = recombinase. Figure A. A20-/- mice genotyping example. Representative genotyping by PCR for identification of K14Crewt/fl_A20wt/fl mice. Expected sizes of products are shown. Figure B Confirmation of localization of KRT14+ cells. Immunohistochemical stainings for expression of KRT14, showing localization in basal layers of excretory, striated, in intercalated ducts and in myoepithelial cells. Figure C No difference was observed in salivary gland infiltration between male and female A20-/- mice. Left panel: Quantification of proportion of total cells staining positive for CD45 in male and female WT and A20-/- mice. n = 3 mice per group per time point. Middle panel: Quantification of proportion of CD3+ cells as proportion of total cells in male and female WT and A20-/- mice. Right panel: Quantification of proportion of B220+ cells as proportion of total cells in male and female WT and A20-/- mice. Figure D No autoantibodies were detected in A20-/- serum. Summary of ANA autoantibody score from WT and A20-/- mice at 30 weeks of age. For scoring system, please see Methods. Figure E Significant weight loss of A20-/- mice necessitates a correction for pilocarpine dose. Top panel: Weights of K14Crewt/fl_A20wt/fl mice compared to WT littermate controls. Bars = S.D. n = ≥ 6 mice per group per time point. Bottom panel: Example corrections of pilocarpine measurements. (DOCX) [file pone.0200212.s001.docx]

# DYSREGULATION OF NF-κB IN GLANDULAR EPITHELIAL CELLS RESULTS IN SJÖGREN’S-LIKE FEATURES

# SUPPLEMENTARY FILE

# Supplementary Materials and Methods

For genotyping and confirmation of A20 gene knockout, ear clippings were taken from mice. To ear clippings, 17.8 µL milliQ water, 2 µL gold buffer and 0.2 µL prepGem was added, and incubated at 75 °C for 15 minutes, followed by 5 minutes at 95 °C. DNA present in the solution was used for PCR detection of genotype. For PCR detection of the A20 gene, 1 µL of each of the P20, P21 and P22 primers (see Supplementary Table 1) were mixed with 6 µL Amplitaq, 14 µL water and 2 µL of DNA. For detection of the Cre gene, 1 µL each of the P13, P38, P39 and P40 primers were mixed with 6 µL Amplitaq, 13 µL water and 2 µL DNA. Samples were incubated at 94 °C for 3 minutes, followed by 40 cycles of the following incubations: 30 sec 94°C, 30 seconds at 55 °C, and 30 seconds at 72 °C. A final incubation at 72 °C for 3 minutes was performed, and samples visualized by gel electrophoresis. An example gel showing A20 and Cre gene status detection is shown in Supplementary Figure 1A.

## Saliva measurements

At 10, 20 and 30 weeks of age, whole stimulated saliva was collected from A20 knockout mice and wildtype (WT) littermate controls. 100 µL of a 0.5 mg / mL solution of pilocarpine hydrochloride (50 µg dose, *aq*) was administered subcutaneously to the animals, and saliva collected by suction pump for 15 min. The quantity of saliva was determined gravimetrically, assuming a density of 1 g / mL saliva. A20^-/-^ mice were significantly smaller than WT controls (Supplementary Figure 3A). The production of saliva in response to pilocarpine stimulation is proportional to the dose received, within dose ranges of approximately 40 µg to 400 µg[8]. To correct saliva data for the size of the A20^-/-^ mice, the dose of pilocarpine (mg) per kg body weight each mouse received was calculated. These doses fell within the proportional ranges of the pilocarpine response curve. Collected saliva volume was adjusted to reflect volume collected if mouse had received the standard dose of 2.5 mg pilocarpine per kg body weight. An example correction calculation can be seen in Supplementary Figure 3B.

## Immunohistochemistry

Mice were sacrificed at 10, 20 and 30 week time points, and submandibular salivary glands harvested. Glands were fixed in 4 % PFA overnight at room temperature (RT), and subsequently dehydrated and cleared for embedding in wax. 4 µM sections of glands were cut, and sections dried overnight. Following dewaxing and rehydration of sections, antigen retrieval was performed by immersion in boiling Sodium Citrate buffer (10 mM pH 6.0 ,containing 0.05 % Tween) for 20 minutes. Endogenous hydrogen peroxide activity was blocked using 0.3 % hydrogen peroxide in PBS (30 minutes, RT), and sections incubated with the following primary antibodies at given concentrations overnight at 4 ˚C: rat anti-mouse CD45 (1:20, BD Biosciences, clone 30-F11), anti-mouse CD3 (1:300, Abcam clone CD3-12), anti-mouse B220 (1:800, BD Pharmingen, clone RA3-6B2). Antibodies were diluted in a buffer containing 1 % bovine serum albumin and 0.05 % Tween. Following washing in PBS, sections were incubated in horse radish peroxidase conjugated secondary antibodies (1:300, Invitrogen) for 1 hour at RT. After washing in PBS, sections were incubated in DAB to visualize staining, lightly counterstained in hematoxylin, and dehydrated and mounted in xylene. Images were captured using the Olympus BX50 microscope and CellB software. A TissueFAxs slide scanning microscope and Histoquest software were used to quantify the percentage of positive cells for each respective marker. Per mouse three sections equally spread through the salivary gland were analyzed. Average number of immune foci (>50 leukocytes together) per 4 mm^2^ of tissue areas was calculated using ImageJ software. Striated ducts containing >1 infiltrating leukocytes, identified by CD45 staining, were enumerated per 4 mm^2^ of tissue area.

## Saliva sample preparation, gel electrophoresis and PAS staining

Frozen mouse saliva samples were thawed, aliquots prepared and stored frozen. The total protein concentration of the salivas was determined using Qubit® 3.0 Fluorometer (Q33216, Invitrogen™, UK), according to the manufacturer’s protocol. For SDS-PAGE, saliva samples were briefly sonicated, then prepared under reducing and heat denatured conditions. Salivary proteins were separated on 4-12% Novex polyacrylamide gels. Running conditions for separation of the high molecular weight proteins were modified as follows: 150 volts (constant), 40mA and 1h. Gels were stained for proteins with Coomassie Brilliant Blue R250 (Sigma-Aldrich, Gillingham, UK) or stained for glycosylated glycoproteins with periodic acid–Schiff (PAS) staining directly on protein gels.

## MUC10 detection by mass spectrometry

Following SDS-PAGE a piece of 4-12% Novex gel known to contain a PAS positive, high molecular glycoprotein was excised and subjected to proteomics analysis by LC MS/MS. Briefly, the gel piece was subjected to trypsin digestion overnight following reduction with DTT. Peptides were extracted and resolved by reverse phase liquid chromatography on a C18 EASY NanoLC system (Thermo Fisher Scientific, UK). The MS/MS analysis was conducted on an Orbitrap Velos Pro system (Thermo Fisher Scientific, UK). Raw mass spectrometry data were processed using Proteome Discoverer v1.4 software (Thermo Scientific) and proteins identified using Mascot search engine software. Five unique peptide sequences were used to identify MUC10. Glycosylated MUC10 quantification was achieved by scan-imaging the PAS-stained gels. Total protein intensities and band intensity at the MUC10 electrophoretic level were calculated using ImageJ software. MUC10 quantification was expressed as: MUC10/total protein for each loaded sample.

## qPCR

Total RNA was extracted from frozen salivary gland tissue sections (15uM thickness, 30 sections per sample). The RNeasy Microkit (Qiagen), including DNase incubation, as per manufacturer’s instructions was used to achieve this. One µg of total RNA was reverse transcribed to cDNA using 0.5 µg oligo(dT)_15-18_ primers, 1.0 mM dNTPs, 1X Reaction Buffer, 20U Ribolock and 200 U of RevertAid Reverse Transcriptase (all Thermo Fischer Scientific), in a total volume of 20 µL per reaction. cDNA product was diluted ten-fold in water and used at this concentration for qPCR. qPCR was performed using SsoAdvanced Universal SYBR Green qPCR Mastermix (Biorad), with primers at a final concentration of 500 nM from a 10 µM stock. 2.0 µL of diluted cDNA was used per reaction, and all reactions were performed in triplicate, in a total volume of 10 µL. A 2-step qPCR cycle with the Applied Biosystems Quant Studio 6 Real-time PCR system was used for target amplification according to SSoAdvanced Universal SYBR Green Mastermix instructions, and Quant Studio Real-time PCR software for analysis. Primer sequences for IFNα, IFNɣ, TNFα, IL6, CXCL10, CXCL13, and GAPDH are listed in Supplementary Table 1.

## Antinuclear antibody screening

Specific ANA were detected following protocol descried in Jan Praet *et al* (2015; EMBO J 12;34(4):466-74). In detail, ANAs were detected by line immunoassay (INNO‐LIA ANA Update, Innogenetics NV). The nylon strips were incubated with serum at a 1:200 dilution. Following washing, a 1:2,500 dilution of an alkaline phosphatase‐conjugated anti‐mouse IgG was added (Chemicon). After washing, the reaction was revealed with the chromogen 5‐bromo‐4‐chloro‐3‐indolyl phosphate, producing a dark brown color in proportion to the amount of specific autoantibody in the test sample. Sulfuric acid was added to stop the color development. The cutoff of the reactivities was determined by testing 20 serum samples from 6‐month‐old C57BL/6 mice as previously described as in van Praet *et al*. None produced any background staining higher than a 1:12,800 dilution of a strong anti‐RNP‐A reactivity of a MRL/lpr^−/−^mouse. Thus, we considered a higher intensity as a positive test result. To determine the intensity of the color as quantification for the amount of antibody in the test sample, further dilutions of the reference anti‐RNP‐A reactivity were used (at least as intense as 1:12,800 was considered 1+, 1:6,400 2+, 1:3,200 3+, 1:1,600 4+, 1:800 5+, and 1:200 6+). The assay contains the following recombinant and natural antigens: SmB, SmD, RNP‐A, RNP‐C, RNP‐70k, Ro52/SSA, Ro60/SSA, La/SSB, CenpB, Topo‐I/Scl70, Jo‐1, ribosomal P, and histones.

# Supplementary Figures

**Table A**

| Primer | Primer sequence | Product size (bp) |
| --- | --- | --- |
| A20 forward (WT & KO) p20 | cacagagcctcagtatcatgt | - |
| A20 KO reverse p21 | cctgtcaacatctcagaagg | 230 |
| A20 WT reverse p22 | gcagctggaatctctgaaatc | 150 |
| Cre WT reverse p13 | gcggtctggcagtaaaaactatc | 400 |
| Cre WT forward p39 | aagatgtggagagttcggggtag |  |
| Cre REC reverse p38 | acgaacctggtcgaaatcagtg | 200 |
| Cre REC forward p40 | gggaccaccttctggcttc |  |
| GAPDH forward | ggagagtgtttcctcgtccc | 202 |
| GAPDH reverse | actgtgccgttgaatttgcc |  |
| IFNα forward | tttcccctgacccaggaaga | 118 |
| IFNα reverse | cttctgctctgaccacctcc |  |
| IFNɣ forward | agcaaggcgaaaaaggatgc | 83 |
| IFNɣ reverse | tcattgaatgcttggcgctg |  |
| TNFα forward | ggagaagggcagttaggcat | 99 |
| TNFα reverse | cccagcaagcatctatgcac |  |
| IL-6 forward | cacttcacaagtcggaggct | 113 |
| IL-6 reverse | ctgcaagtgcatcatcgttgt |  |
| CXCL10 forward | agtgctgccgtcattttctg | 130 |
| CXCL10 reverse | tccctatggccctcattctca |  |
| CXCL13 forward | ctctctccaggccacggtat | 206 |
| CXCL13 reverse | tgtaaccatttggcacgagga |  |

**Fig A.**


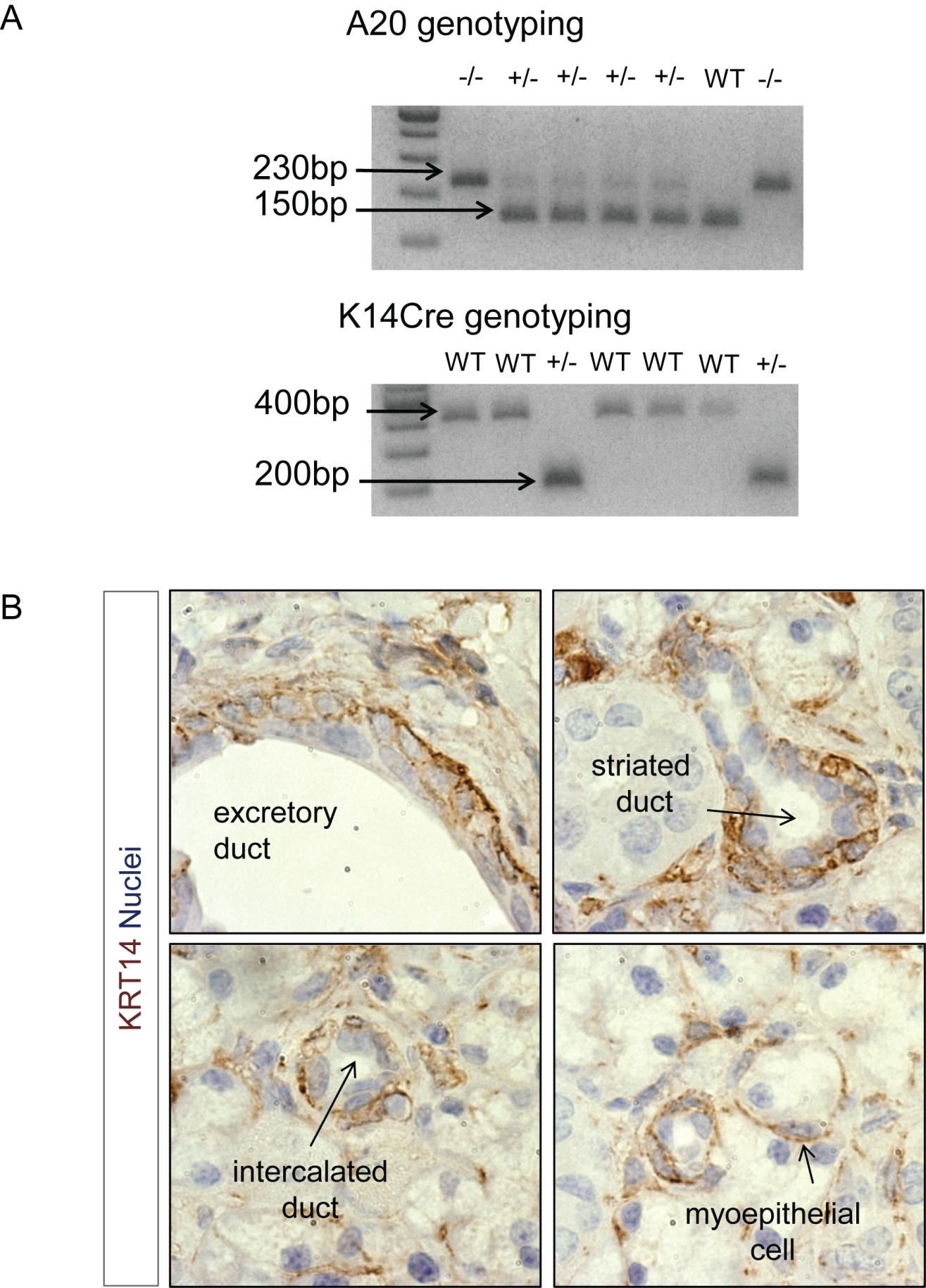


**Fig B**


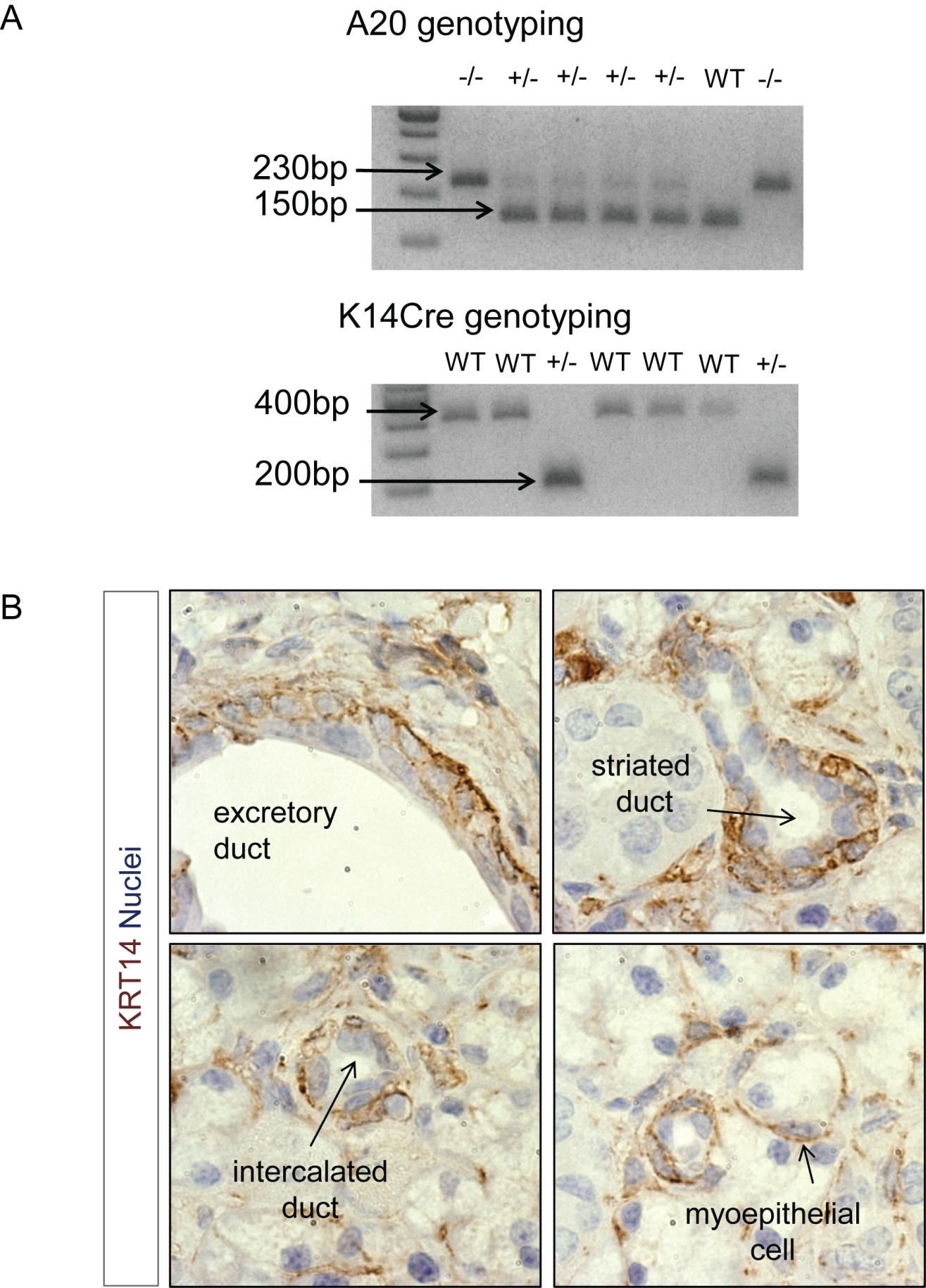


**Fig. C**

**
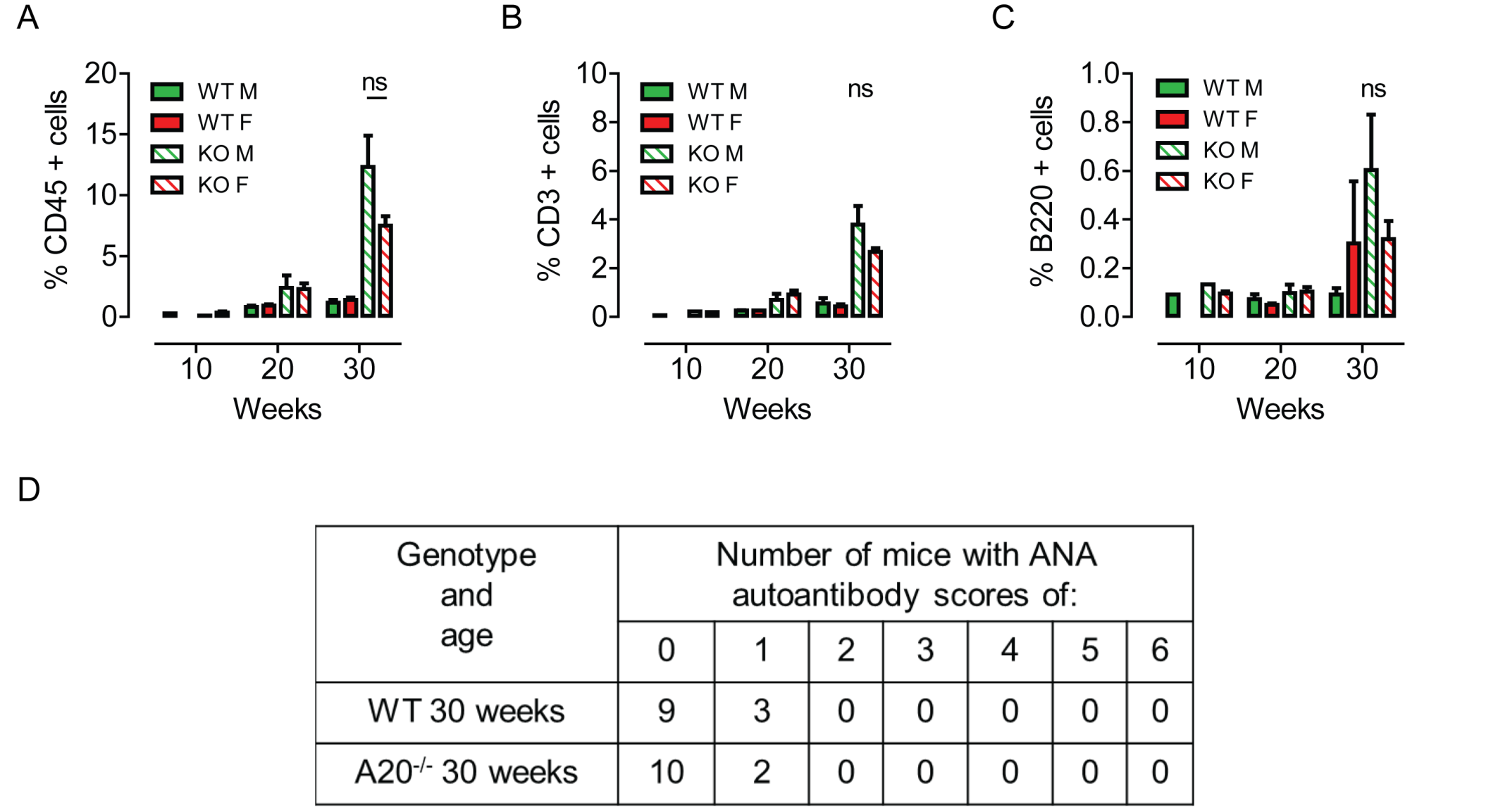
**

**Fig D.**

**
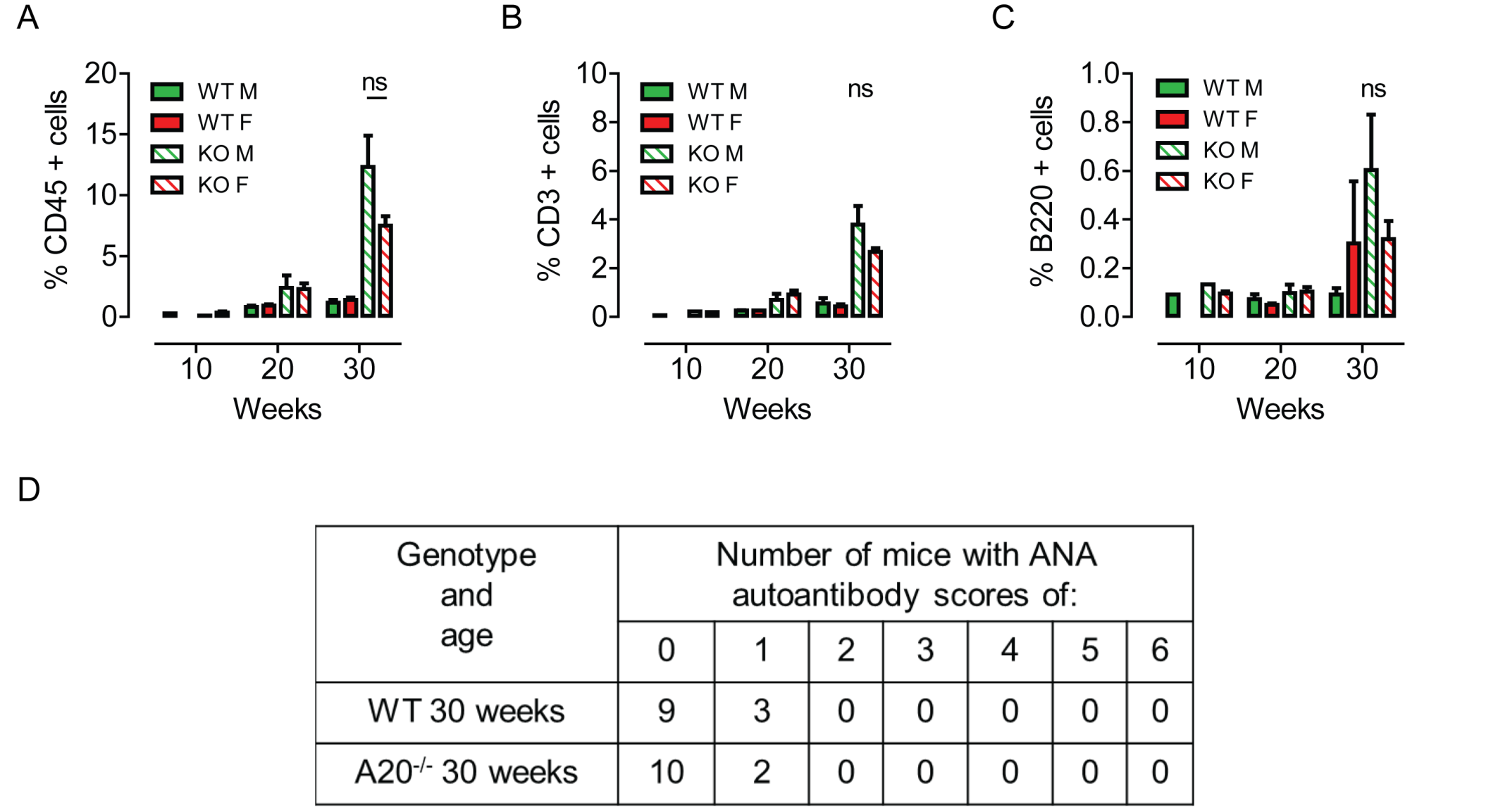
**

**Fig. E**


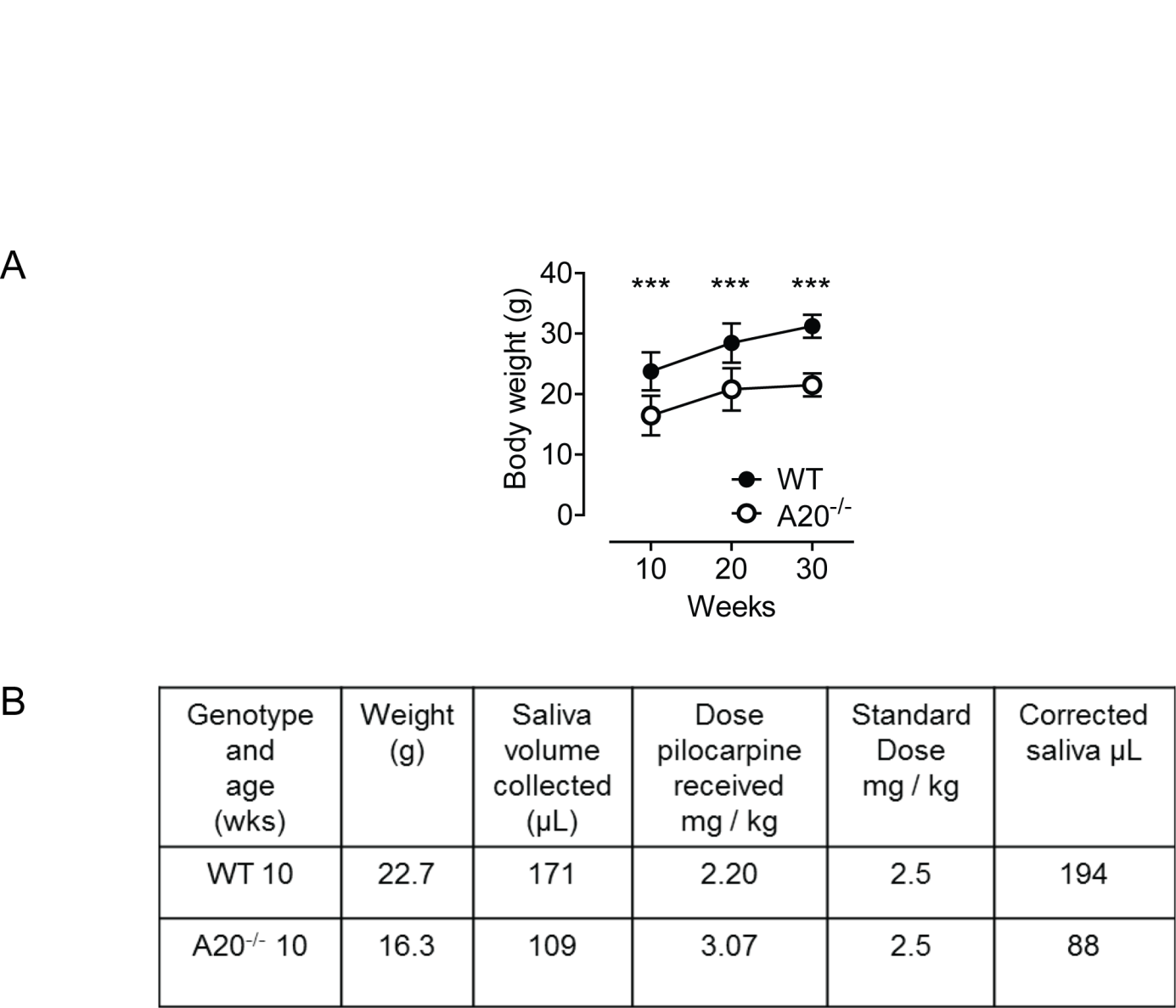


| Genotype  and  age  (weeks) | Weight  (g) | Saliva volume collected (µL) | Dose pilocarpine received  mg / kg | Standard dose  mg / kg | Corrected saliva  µL |
| --- | --- | --- | --- | --- | --- |
| WT 10 | 22.7 | 171 | 2.20 | 2.5 | 194 |
| A20-/- 10 | 16.3 | 109 | 3.07 | 2.5 | 88 |
